# The Relationship of the Psychological Resilience and Stress Coping Level of Health Personnel Working in Hospitals After Kahramanmaraş-Based Earthquakes with Traumatic Stress

**DOI:** 10.3390/healthcare13030301

**Published:** 2025-02-02

**Authors:** Mehmet Koca, Serdar Deniz, Feyza İnceoğlu

**Affiliations:** 1 Department of Health Management, Malatya Turgut Özal University, Malatya 44210, Türkiye; 2 Department of Public Health, Malatya Turgut Özal University, Malatya 44210, Türkiye; serdar.deniz@ozal.edu.tr; 3 Department of Biostatistics, Malatya Turgut Özal University, Malatya 44210, Türkiye; feyza.inceoglu@ozal.edu.tr

**Keywords:** earthquake, trauma, stress, psychological resilience, health workers

## Abstract

Objectives: In this study, we aimed to examine the indirect and direct effects of the stress experienced by health workers after an earthquake on their psychological resilience. Methods: This study was conducted using the Structural Equation Model on 315 employees serving in hospitals in Kahramanmaraş, Gaziantep, Şanlıurfa, Diyarbakır, Adana, Adıyaman, Osmaniye, Hatay, Kilis, Malatya, and Elazığ (11 provinces) that were most affected by the earthquake. Results: The study group consisted of 58.4% (*n* = 184) women and 41.6% (*n* = 131) men. The mean score on the Psychological Resilience Scale was 71.74, the mean score on the Coping with Stress Scale was 87.17, the mean score on the Traumatic Stress Symptom Scale was 44.04, and the mean score on the Disability Scale was 3.75. Psychological resilience had a statistically significant negative effect on traumatic stress symptoms (β_1_ = −0.26; *p* = 0.001 < 0.05). There was a statistically significant positive relationship between coping with stress and psychological resilience (β_1_ = 0.52; *p* = 0.001 < 0.05). Conclusions: It was concluded that an increase in an individual’s psychological resilience will increase the level of coping with stress, and the traumatic stress symptoms of individuals with an increased level of coping with stress will decrease.

## 1. Introduction

Two earthquakes centered in Kahramanmaraş occurred in Türkiye on 6 February 2023, approximately nine hours apart and classified as quite strong according to the Richter scale. The epicenters of these earthquakes were in the Pazarcık and Ekinözü districts of Kahramanmaraş. According to the United States Geological Survey (USGS), the magnitude of the earthquake was given as 7.8 and 7.5, respectively. In our country, Türkiye, the magnitude of the earthquake was given as 7.7 and 7.6, according to the Disaster and Emergency Management (AFAD) and Boğaziçi University and Kandilli Observatory Earthquake Research Institute [1].

As a result of the earthquakes, at least 50,783 people died in Türkiye, 8476 people died in Syria, and more than 122 thousand people were injured in total according to official figures [2]. The earthquake was felt over a wide geographical area, including Türkiye and Syria, as well as Lebanon, Cyprus, Iraq, Israel, Jordan, Iran, and Egypt. It caused damage over an area of approximately 350,000 km^2^. Approximately 16% of the Turkish populations, i.e., 14 million people, were affected by the earthquake. The first earthquake in Pazarcık with a magnitude of 7.8 Mw was the second strongest earthquake in Anatolia after the North Anatolian earthquake of 1668, with an estimated magnitude of 7.8 to 8.0, and the largest earthquake in the Republic of Türkiye on the surface wave magnitude scale. The second earthquake, which occurred 9 h later in Ekinözü with a magnitude of 7.5 Mw, was the third strongest earthquake in Turkey and the deadliest earthquake in the world since the 2010 Haiti earthquake, which killed more than 300,000 people [3].

There are many definitions of a disaster. The United Nations (UN) defines disasters as “all kinds of natural, technological or human-induced events that cause loss of human life and property, affect the society physically, psychologically and economically and cannot be dealt with local opportunities” [4].

As disasters occur over a wide area and the number of people affected by these disasters is high, many people are injured, lose their lives, become disabled, and also experience psychological problems. Disasters also increase the need for emergency health services and threaten social infrastructure. Sometimes, these effects can last a long time [5]. Disasters often cause more problems than the city or country in which they occur can cope with [6,7].

Due to the nature of disaster response, rescuers, especially healthcare workers, are at risk of psychological problems, including acute stress disorder, anxiety and depression, post-traumatic stress disorder, and other psychological disorders [8]. The concept of trauma is defined as the impact of sudden and unexpected life events. Events that have devastating consequences on individuals were defined as “trauma” for the first time in DSM-III. In DSM V, while in “primary trauma” individuals are directly exposed to a painful traumatic experience themselves, “secondary trauma” is used to describe the situation experienced by individuals in various difficulties when they witness exposure to trauma. The negative effects of secondary trauma are thought to be quite similar or even identical to the reactions to primary exposure to trauma [9,10]. Post-traumatic stress disorder is also explained by different concepts, such as compassion fatigue, indirect traumatization, and secondary traumatic stress [11]. Post-traumatic stress disorder is a disorder characterized by persistent intense reactions to reminders of a traumatic event, mood swings, a sense of imminent threat, sleep disturbance, and hypervigilance [12].

Stress is defined as a state of physical or psychological overstimulation of the body due to an individual seeing stimuli they are exposed to in their internal and external environments as harmful to themself [13].

Psychological resilience can be expressed as an individual’s ability to adapt to their environment when faced with negative events. Individuals with high psychological resilience are less likely to have mental illnesses. A person’s ability to cope with negative situations and adapt to difficult situations is an indication that they have a strong personality. The most prominent feature of these people is the ability to manage and direct their lives. Individuals having this power increase their self-confidence. Their openness to knowledge and experience enables them to participate in life actively, and their positive attitude toward change and transformation contributes positively to their lives. Individuals who can manage their emotions and have high emotional intelligence are skilled in coping with a calm and positive approach to events [10,14,15].

According to Folkman and Lazarus, the process of coping with stress is the use of coping resources by appraising the stressful situation. Physical, emotional, and mental strategies play an important role in coping with stress [16].

Gioastra et al. (2025) examined the relationship between emergency stress, secondary trauma, and burnout in their study on Red Cross volunteers working in the COVID-19 pandemic and found a strong positive relationship between emergency stress, secondary trauma, and burnout and found that it had a negative correlation with resilience skills [17]. Again, Maiorano et al. (2020) concluded in their study on health and emergency workers during the COVID-19 pandemic that nurses and doctors experienced higher levels of emergency stress than emergency workers and that coping strategies and resilience were protective factors and reduced the impact of stress on secondary trauma [18].

Healthcare professionals living in the disaster-affected area not only serve as rescuers but are also affected by the disaster themselves, losing their families and friends and having difficulty communicating with them. In addition to their increasing workload, they are unable to meet their daily needs and experience mental fatigue, and these have negative effects on mental health [19,20].

In their study conducted after the earthquakes in Türkiye, Bulut et al. (2023) found a significant difference between post-traumatic stress disorder in healthcare workers who worked and did not work in the earthquake region [21]. Yanık and Ediz (2024), in their study on nurses who provided voluntary care in the earthquake region, stated that nurses were deeply affected by the psychosocial aspects of the disaster and often struggled with inadequacies in coping with psychological difficulties [22]. Altuntaş et al. (2023) reported that it is significant to utilize the knowledge of nurses about disaster experiences to provide more effective healthcare services in disaster periods and to identify the problems experienced to guide future planning processes [23].

In crises such as earthquakes, healthcare professionals prioritize the needs of their patients over their own needs. They are exposed to conditions such as weakened healthcare infrastructure, insufficient medical supplies, long working hours, staff shortages, and difficulties in accessing basic supplies, as well as mental fatigue, physical fatigue, anxiety, burnout, and post-traumatic stress disorder [24]. Investigating resilience and related factors has become increasingly important in studies examining the occupational mental health of healthcare workers [25].

In this context, it is expected that the psychological resilience of health personnel who continue to work in harsh conditions after the earthquake will play an active role in their coping with stress. Depending on their psychological resilience, their level of coping with stress will allow them to manage the traumatic stress they have experienced after the earthquake. In this process, psychological resilience will function as a tool, play an active role in emotional balance, and increase the effectiveness of stress management. The psychological state of individuals is an important factor in managing secondary traumatic stress [10,15]. Individuals with good stress coping mechanisms play a more effective role in the management of traumas experienced during the earthquake process [26]. In extraordinary situations such as earthquakes, the healthcare professionals in the region who provide the first intervention for injured people and then have to provide treatment for a longer period of time may be exposed to secondary trauma in addition to experiencing primary trauma. The main purpose of this study was to examine the mediation effect of psychological resilience on the relationship between the levels of coping with stress and the post-earthquake traumatic stress of health personnel working in provinces affected by the earthquake. Thus, first, the post-earthquake traumatic stress levels, psychological resilience, and stress coping profiles of the health workers were revealed. In the second stage, the relationships between psychological resilience and post-earthquake traumatic stress situations were examined, and the mediating effect of coping with stress levels on this relationship was analyzed.

## 2. Materials and Methods

### 2.1. The Type and Hypotheses of the Research

This study was designed using a relational screening methodology. The purpose of employing the relational screening model is to analyze the interactions among two or more sets of variables using various methods (both direct and indirect effects) [27]. In this study, the mediating effect of coping with stress on the relationship between traumatic stress symptoms and psychological resilience was investigated.

The working hypotheses are stated below:

**H_1_:**  *Traumatic stress symptoms are statistically significant in reducing psychological resilience scores*.

**H_2_:**  *Traumatic stress symptoms are statistically significant in reducing coping with stress scores*.

**H_3_:**  *Psychological resilience is statistically significant in increasing coping with stress scores*.

**H_4_:**  *Coping with stress has a mediating effect on the relationship between traumatic stress symptoms and psychological resilience*.

### 2.2. The Place and Time of the Research

This study was conducted via face-to-face interviews and Google Forms with healthcare professionals employed in hospitals across eleven provinces in Türkiye—Kahramanmaraş, Gaziantep, Şanlıurfa, Diyarbakır, Adana, Adıyaman, Osmaniye, Hatay, Kilis, Malatya, and Elazığ—that were significantly impacted by the earthquake and had declared a state of emergency between 20 April and 15 May 2023. During the data collection phase, the forms were restricted to a single response from each participant. Cookies and IP addresses were examined to verify data integrity.

### 2.3. Sample Selection and the Number of Samples

While a precise definition for the Structural Equation Model (SEM) is lacking, Schumacker and Lomax (2004) noted that certain studies employ sample sizes ranging from 250 to 500 [28]. Kline (2011) asserts that analyses utilizing a SEM necessitate a sample size of 200 or above. In line with these opinions, 315 participants were recruited for this study [27]. The participants were selected using snowball sampling methods, which are based on voluntariness, which is one of the nonprobability sampling methods.

### 2.4. Data Collection Tools

#### 2.4.1. Personal Information Form

The authors developed a 27-question sociodemographic questionnaire to gather information on age, gender, marital status, education, province of employment, health status, loss of a relative after the earthquake, and damage to one’s residence.

#### 2.4.2. The Psychological Hardiness Scale (PHS)

Işık (2016) proposed a scale consisting of 21 questions in three sub-dimensions, a validity and reliability analysis of which was undertaken, and prepared as a 5-point Likert format. Higher scores on the PHS indicate higher levels of resilience [14].

#### 2.4.3. The Coping Responses Inventory (CRI)

This was developed by Moos in 1993 and translated into Turkish by Koca Ballı and Kılıç in 2016 for validity and reliability. The scale has five sub-dimensions: logical analysis, positive evaluation, seeking professional support, seeking environmental support, and problem solving. The scale is prepared as a 5-point Likert scale and consists of 24 questions [29,30].

#### 2.4.4. The Post-Earthquake Traumatic Stress Screening Scale (PETSSS)

The Post-Earthquake Traumatic Stress Screening Scale was created by Başoğlu et al. (2001) as a result of their studies after the Marmara and Düzce earthquakes. The scale consists of three parts. In the first part, there is the “Information Form in the Earthquake”, in the second part, the “Traumatic Stress Symptom Scale (TSSS)”, and in the third part, the “Disability Scale (DS) Form”. In our research, the “Traumatic Stress Symptom Scale (TSSS)” and the “Disability Form” were used in the third section. The “Traumatic Stress Symptom Scale (TSSS)” of the questionnaire consists of 17 questions and is structured in a 4-point Likert style. It was created to determine whether people have experienced certain problems in the last week, and if they have, to what extent these problems have bothered them. The scores of the items in the scale range from 0 to 3 (0 = not at all disturbing, 1 = slightly disturbing, 2 = quite disturbing, 3 = very disturbing), and the scale is in the range of 0–51 points in total. On the other hand, the “Disability Form” asks three questions to determine the disorders caused by the TSSS symptoms. In the first question, respondents are asked to tick the appropriate option to determine how much they are bothered by the issues on the PTSS scale (0 = never, 1 = slightly, 2 = quite a bit, 3 = severely), and in the second question, they are asked to determine the degree to which these issues affect their work, family, and social life (0 = no problem, 1 = bothers me a little, 2 = bothers me a lot, 3 = is severely disruptive). The last question in the questionnaire is used to determine whether the person has sought help from a doctor or psychologist due to their mental state (0 = no, 1 = yes, 2 = I am not sure, I do not know) [31].

### 2.5. Ethics Approval

Ethical approval for this investigation was granted by the noninterventional clinical research ethics committee of Malatya Turgut Özal University, as documented in the letter dated 12 April 2023, with decision number E-30785963-020-154279. This research was performed in compliance with the principles of the Declaration of Helsinki. Consent was secured from the participants.

### 2.6. Statistical Analysis

The research data were analyzed using SPSS (Statistical Package for the Social Sciences) version 25. Descriptive statistics of the variables were presented using numerical values, percentages, means, and standard deviations.

The Cronbach’s α coefficient was employed to assess the reliability of the scales. The SEM analysis was conducted using the AMOS 24 software suite. Prior to the multivariate analysis, the data were assessed for a multiple normal distribution utilizing the “Observations Farthest from the Centroid (Mahalanobis Distance) Menu” in the AMOS software. The model’s skewness value was computed as 2.389, and as it was below 8, a multivariate normal distribution was attained [32].

To ascertain the link between the independent variables and the scales, a linear correlation analysis was conducted [33].

A mediated SEM analysis using the bootstrap approach was conducted on the study data using the AMOS 24 software. The bootstrap approach is posited to be more dependable than the classical Baran and Kenny method and the results derived from the Sobel test. To execute the bootstrap procedure, 5000 samples were resampled. If the lower and upper limits of the 95% confidence interval derived from the bootstrap approach do not encompass the value zero (0), it is concluded that a mediation effect exists between the variables [34].

Nonparametric testing methods were employed to compare the variables in the study. The Mann–Whitney U test was employed to compare variables between two groups, whereas the Kruskal–Wallis test was utilized for comparisons involving more than two groups. The Mann–Whitney U test with Bonferroni correction was conducted for post hoc comparisons of variables that exhibited differences following the Kruskal–Wallis test.

## 3. Results

### 3.1. Demographic Information

The demographic information of the participants included in this study is given in the table below.

This study was conducted with n = 315 participants. A total of 58.4% (n = 184) of these participants were women. A total of 69.5% (n = 219) of the participants were married, 71.7% (n = 226) had a child, and 40% (n = 126) had a bachelor’s degree. Again, 51.1% of the participants stated that they were healthcare workers, 29.5% (n = 93) had 21 years or more of working time, 70.2% (n = 221) did not have any chronic illness, 84.8% (n = 267) did not have a disabled person or an individual over the age of 80 in their household, and 77.1% (243) did not pay loans for their homes (Table 1).

Of the participants, 62.9% (n = 198) had not received disaster training, 84.4% had not lost a relative to the earthquake, 65.1% (n = 205) owned the house they lived in, 65.7% (n = 207) had an undamaged or slightly damaged house, 41.3% (n = 130) stayed in their own home after the earthquake, 60.3% (n = 190) did not send their family members out of town after the earthquake, 70.2% (n = 221) did not want to be transferred or temporarily assigned outside the disaster area, 66.4% (n = 209) stated that their working order changed after the disaster, 67% (n = 211) had difficulty accessing basic necessities after the disaster, 57.8% (n = 182) did not have compulsory earthquake insurance at home, 76.5% (n = 241) stated that they did not have housing insurance, and 29.9% (n = 94) of those whose houses were moderately damaged or severely damaged could not access all or some of their belongings (Table 2).

### 3.2. Descriptive Statistics of the Scale Scores

The descriptive statistics and Cronbach’s α reliability coefficients for the traumatic stress symptoms (traumatic stress and disability), psychological resilience, and stress coping scores used in this study are given in Table 3.

The scales employed exhibited elevated Cronbach’s α values of 0.70 for traumatic stress symptoms, disability, psychological resilience, and stress coping, indicating strong reliability [35].

The mean score on the Psychological Hardiness Scale was 71.74 ± 11.1, the mean score on the Coping with Stress Scale was 87.17 ± 12.66, the mean score on the Traumatic Stress Symptom Scale was 44.04 ± 11.9, and the mean score on the Disability Scale was 3.75 ± 2.2.

As a result of the analysis, a statistical difference was found between men and women in their CRI, TSSS, DS, and PETSSS scores (*p* < 0.05). However, no statistical difference was found between the men and women in their PHS scores (*p* > 0.05). No statistical difference was found between healthcare workers and administrative workers in their PHS, CRI, TSSS, DS, and PETSSS scores (*p* > 0.05). A statistical difference was found in the PHS, CRI, TSSS, DS, and PETSSS scores according to years of employment (*p* < 0.05). In the post hoc comparisons of the PHS, CRI, TSSS, DS, and PETSSS scores, a statistical difference was found between those who had worked for less than 5 years and those who had worked for 11–15 years and between those who had worked for 6–10 years and those who had worked for more than 20 years (*p* < 0.05) (Table 4).

Before the SEM analysis, firstly, the relationships between the scales and the changes in the age variable according to the scale scores were tested, and the results are given in Table 5.

No statistically significant relationship was found between age and PHS, CRI, TSSS, DS, and PETSSS score (*p* > 0.05).

A positive moderate correlation was found between the PHS and CRI scores (r = 0.520); a negative low correlation was found between the TSSS (r = −0.281), DS (r = −0.270), and PETSSS (r = −0.202) scores; a negative low correlation was found between the CRI and TSSS (r = −0.266), DS (r = −0.241), and PETSSS (r = −0.284) scores; a positive high correlation was found between the TSSS and DS (r = 0.749) and PETSSS (r = 0.994) scores; and a positive high correlation was found between the DS (r = 0.749) and PETSSS (r = 0.816) scores (*p* < 0.05).

### 3.3. The Mediation Analysis

**Figure 1:**
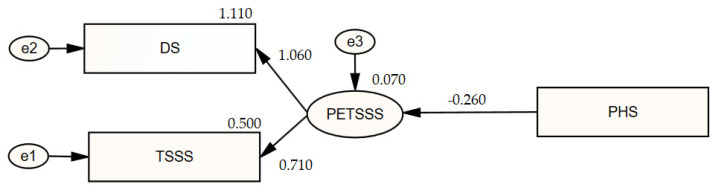
Diagram of the measurement model illustrating the link between traumatic stress symptoms and psychological resilience. PHS: Psychological Hardiness Scale; CRI: Coping Responses Inventory; TSSS: Traumatic Stress Symptom Scale; DS: Disability Scale; PETSSS: Post-Earthquake Traumatic Stress Screening Scale.

The SEM was developed to assess the mediating role of stress coping in the link between traumatic stress symptoms and psychological resilience. Initially, a measurement model was developed to assess the correlation between traumatic stress symptoms and psychological resilience, followed by an examination of the link between the scales. The measuring model is illustrated in the graphic presented in Figure 1.

In the model, psychological resilience serves as the independent variable, traumatic stress symptoms function as the dependent variable, and e1, e2, and e3 represent the residual terms. The model’s coefficients are presented in Table 6.

Psychological resilience had a statistically significant negative effect on traumatic stress symptoms (β_1_ = −0.26; *p* = 0.001 < 0.05). If a person’s psychological resilience increases by 1 point, their traumatic stress symptom score will decrease by 0.20 points (β_1_= −0.20). The psychological resilience score explained 7% of the change in the traumatic stress symptom score (R^2^ = 0.07).

**Figure 2:**
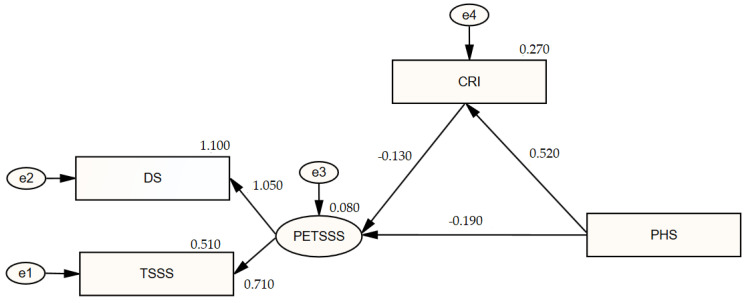
Diagram of the mediating effect of coping with stress on the relationship between traumatic stress symptoms and psychological resilience. PHS: Psychological Hardiness Scale; CRI: Coping Responses Inventory; TSSS: Traumatic Stress Symptom Scale; DS: Disability Scale; PETSSS: Post-Earthquake Traumatic Stress Screening Scale.

Since the measurement model was significant, the model of the mediating effect of coping with stress in the relationship between traumatic stress symptoms and psychological resilience was established, and the model diagram is given in Figure 2.

Table 7 presents the regression coefficients and significant values that aid in interpreting the analysis results pertaining to the mediation model.

In the mediation model, stress coping scores serve as the dependent variable for psychological resilience and as the independent variable for the traumatic stress symptom variable. In structural equation modeling analyses including several simultaneous regression models, a variable may function as both a dependent and an independent variable concurrently. Structural equation modeling analyses provide researchers with enhanced interpretative capabilities [36].

A statistically significant positive relationship exists between stress coping mechanisms and psychological resilience (β_1_ = 0.52; *p* = 0.001 < 0.05). If psychological resilience grows by 1 point, the stress coping score will rise by 0.59 points (β_2_ = 0.59). Psychological resilience accounted for 27% of the variance in the stress coping scores (R^2^ = 0.27).

A statistically significant negative relationship was observed between traumatic stress symptoms and psychological resilience (β_1_ = −0.14; *p* = 0.008 < 0.05), as well as dealing with stress (β_1_ = −0.09; *p* = 0.048 < 0.05). The traumatic stress symptom score will diminish by 0.14 (β_2_ = −0.14) points with a 1-point rise in psychological resilience and by 0.09 (β_2_ = −0.09) points with a 1-point increase in stress coping. The scores for stress coping and psychological resilience accounted for 8% of the variance in the traumatic stress symptoms (R^2^ = 0.08). The evaluation of the bootstrapping data, a novel method for mediation analysis, revealed that the indirect effect of stress coping on traumatic stress symptoms and psychological resilience was statistically significant (β = −0.068; CI [0.014–0.04]). The mediating influence of stress coping on the association between traumatic stress symptoms and psychological resilience was assessed using the percentage technique. The bootstrap lower confidence interval limit (CIlower = 0.014) and the upper confidence interval limit (CIupper = 0.04) were determined to exclude the value zero (0). The mediation model constructed was statistically significant, as the confidence interval did not encompass 0. The unstandardized regression coefficient between traumatic stress symptoms and psychological resilience in the measurement model (−0.26) was observed to diminish in the mediation model (−0.19). The correlation between the two scales diminished upon the incorporation of the mediating variable into the model, and a partial mediation effect was computed [37,38].

Structural equation modeling assesses all indices collectively rather than relying on a singular fit index to determine the validity of the research-derived model. The new model yielded a goodness-of-fit index score of χ^2^/df (Chi-square Goodness of Fit; χ^2^, degrees of freedom) of 1.012. The RMSEA (Root Mean Square Error of Approximation) score, indicating sample adequacy, is 0.073 (RMSEA < 0.80), signifying that the sample size is enough for the employed model. The model demonstrates an excellent fit, as indicated by the GFI (0.998), CFI (0.993), IFI (0.993), and NFI (0.997), with all exceeding the threshold of 0.90 for fit indices [36].

## 4. Discussion

People encounter events or disasters that traumatize them at various levels throughout their lives. Not knowing where, when, and how strong an earthquake, which is one of these kinds of disasters, will occur increases stress and anxiety in people. It can be said that large earthquakes are disasters that traumatize individuals due to their large impact area and destructive power. It has been stated that the stress symptoms that will occur due to trauma, especially after an earthquake, are between 3 and 87% in population segments with various sociodemographic characteristics [39]. It is not possible to explain this increase only using various individual independent variables. The development level of the country, the duration of the earthquake, the destruction it causes, deaths, and many other cultural factors play a role [40,41].

The scope of a disaster and a group’s exposure to this disaster are likely the most important factors determining the ultimate prevalence of PTSD [42]. In their study on rescue team workers in the earthquake that occurred in Pakistan in 2005, Ehring et al. (2011) found that people needed shelter (41.9%) and food and water assistance (39.3%), had to deal with their own problems, their homes were damaged (37.1%), and even had to move (30%) [43]. In this study, 15.6% of the participants lost a relative in the earthquake. Since the loss of relatives is an irreparable situation, it is among the most important factors that increases stress in individuals. It was also determined that 34.3% of the participants’ homes suffered moderate or higher damage from the earthquake, making them uninhabitable; 58.7% of them were living in tents, containers, etc., outside the home that they lived in before the earthquake; and 60% experienced varying degrees of difficulty accessing basic necessities. This becomes even more challenging for those with chronic illnesses (29.8%) and for those with people with disabilities or over 80 years of age for whose care they are responsible in their households (15.2%). These rates show that the earthquake healthcare workers had varying degrees of challenges accessing shelter and basic living materials, especially until help arrived from other regions.

Established in Türkiye in 2000, the Natural Disaster Insurance Institution (NDII) is a public institution with a legal identity responsible for the acquisition, implementation, and management of compulsory earthquake insurance in our country. Compulsory earthquake insurance is a coverage system that covers the structural damage (e.g., the main walls, garden walls, ceiling and elevator failures, and original elements of the structure, such as plaster, roof, and ceramics) that occurs in the residence due to an earthquake. This coverage system does not cover used household items, such as sofas, televisions, refrigerators, and washing machines. It is also necessary to have home insurance to cover your household goods. Although compulsory earthquake insurance is mandatory in Türkiye, unfortunately, most people do not have this insurance. The rate of having compulsory earthquake insurance is around 56%. The compulsory earthquake insurance rate in earthquake zones is around 50% [44].

When the insurance status of the participants in this study was examined, it was determined that the rate of those who had insured their homes with compulsory earthquake insurance was 42.2%. The rate of those who had home insurance was 23.5%. The proportion of the participants who also paid a home loan was 22.9%. The rate of those who could not retrieve their belongings from their damaged houses was 15.9%, and the rate of those who were able to retrieve some of their belongings was 14%. These rates show that the majority of the healthcare workers affected by the earthquake also experienced financial losses at various levels. The biggest loss due to an earthquake is the loss of loved ones. However, the loss of homes and belongings due to the earthquake is also thought to play an important role in increasing the level of post-traumatic stress disorder. This view is supported by a study conducted among adolescents affected by the Wenchuan earthquake in China, where loss of one’s home and property, injury, the death of family members, and witnessing death were considered positive risk factors for post-traumatic stress disorder [45].

Bedirli (2014) conducted a study on the people affected by the Gölcük and Düzce earthquakes in 1999 and stated that 18.1% of the participants migrated [46]. In their study, Tovaranonte and Cawood (2013) found that 8% and 35% of families moved to another city after the earthquakes that occurred in and around the city of Christchurch on 4 September 2010 and 22 February 2011, respectively [47]. In our own study, the rate of those who sent their relatives out of the province was 36.2%. At the same time, it was determined that 29.8% of the employees requested assignment to other regions. This situation is thought to be due to the psychological effects of the earthquake and the time it will take to rehabilitate the areas affected by the earthquake. Rapid and widespread community support is essential to reduce this rate and prevent people from leaving their home areas.

Similarly, in the study conducted by Bedirli (2014), the average score on the TSBÖ was 31.9 [46]. In our own study, the standard deviation was 44.04 ± 11.9. Thus, it seems that the average results of studies conducted using the same scale are different. This difference is thought to be because the study by Bedirli (2014) was carried out long after the earthquake, a long period of time had passed after the trauma, and the impact area and size of the two earthquakes were different.

Studies conducted by Pak et al. (2017) on emergency service workers, by Erdener (2019) on disaster workers, by Cebbar (2021) on psychologists, and by Polat (2022) on healthcare workers found that there is a significant negative relationship between psychological resilience (PR) and secondary traumatic stress [10,15,48,49]. Vogt et al. (2008) and Işık (2016) stated that people with high levels of psychological resilience can cope with stress more easily and adapt to stressful situations more easily and protect their mental and physical health [14,50]. The results of this study, similar to the results of the studies above, showed a statistically significant negative effect of psychological resilience on traumatic stress symptoms (β_1_ = −0.26; *p* = 0.001 < 0.05). It was found that increases in traumatic stress symptoms cause negative effects on individuals’ psychological well-being.

In a study conducted by Tsuno et al. with local government employees after the Great East Japan Earthquake, which caused few injuries and material damage, a relationship was found between psychological resilience and post-traumatic stress symptoms [51]. In a 2015 study conducted with 291 people after the Nepal earthquake, it was observed that earthquakes caused negative psychological effects on people, post-traumatic stress levels were high, and negative coping strategies negatively affected TSSS scores [52].

In the research by Çoban (2020) regarding search and rescue personnel in emergency assistance and catastrophes in Istanbul, the correlation coefficient between the average scores of the Coping with Stress Scale and the average scores of the Psychological Resilience Scale was 0.584 [53]. This study corroborates Çoban’s (2020) findings, revealing a statistically significant positive correlation between stress coping and psychological resilience (β_1_= 0.52; *p* = 0.001 < 0.05). It was established that a 1-point increase in psychological resilience corresponds to a 0.59-point increase in the stress coping score (β_2_ = 0.59). The mediation effect analysis model demonstrated that higher levels of stress coping correlate favorably with psychological well-being.

In a study conducted by Aytekin (2003) on hospital employees, 54.4% of the employees stated that they participated in disaster-related training [54]. In the results of our study, the rate of those who received in-service training on disasters was 37.1%. In a study conducted by Dinçer (2019) on healthcare personnel working in Istanbul Medipol University Hospital, it was found that the disaster preparedness of the group that received disaster-related training before was statistically significant compared to that in the group that did not receive disaster-related training [55]. Hence, it is significant to provide training that will contribute to psychological readiness before disasters to ensure the continuation of functionality during the delivery of the service after the disaster [56].

Tommasi et al. (2024) illustrated the significance of psychological education and peer support in their research on the prevention of trauma and sorrow in emergency and critical care settings. Psycho-education can enhance awareness of the psychological impacts of trauma and distressing situations; however, peer support underscores the significance of sharing thoughts and feelings among colleagues immediately following the events as a crucial intervention for mitigating traumatic emotions [57]. Based on the conducted studies, training should be implemented to enhance the psychological resilience of workers during the pre-disaster preparedness phase, with subsequent training sessions following the crisis, emphasizing the need to foster colleague and social solidarity.

In this study, no statistically significant relationship was found between age and PHS, CRI, TSSS, DS, and PETSSS scores. Erdener (2019) found a significant positive relationship between age and psychological resilience in his study and stated that psychological resilience increases with increasing age. He concluded that there was an inverse correlation between age and secondary traumatic stress [15]. However, in some other studies, no significant relationship was found between age and psychological resilience [53,58]. In another study, no significant relationship was found between age and traumatic stress [59].

In this study, no significant difference was found between PHS score and gender. This result is similar to the results of other studies in the literature [15,53]. However, a significant relationship was found between gender and CRI, TSSS, DS, and PETSSS scores. According to the results of the study, it was determined that women generally had higher levels of post-earthquake traumatic stress and their levels of coping with stress were lower than those in men. It is thought that the duties undertaken by women socially and their psychology affect this.

The hypothesis of the negative effect of traumatic stress symptoms on psychological resilience (H_1_), the hypothesis of the negative effect of traumatic stress symptoms on coping with stress (H_2_), the hypothesis of the positive effect of psychological resilience on coping with stress (H_3_), and the hypothesis of the mediating effect of coping with stress in the relationship between traumatic stress symptoms and psychological resilience (H_4_) were statistically significant according to the established mediation model.

## 5. Conclusions

This study revealed that psychological resilience and stress coping scale scores significantly influenced the Traumatic Stress Symptom Scale score. The model indicated that stress coping worked as a mediator between psychological resilience and symptoms of severe stress. An increase in an individual’s psychological resilience will increase their level of coping with stress, and the traumatic stress symptoms in individuals with increased levels of coping with stress will decrease. The hypotheses established for this study were statistically significant and were not rejected.

People do not always encounter major disasters; they are perhaps exposed to disasters once in their lives. Therefore, being prepared for disasters and knowing what to do before, during, and after a disaster are only possible with education. Knowing how to act during a disaster reduces people’s stress and trauma levels. Considering that the rate of receiving disaster-related education is extremely low, it would be useful to review the duration, content, and form of the education given. Again, social support activities are important for people to cope with the stress they face in unexpected situations such as disasters and to cope with these problems. In this framework, it is recommended that social support mechanisms be implemented quickly in stressful situations such as disasters, that the necessary trained professional human resources are always ready for this, and that the necessary mechanisms and institutional structures be established and existing ones be strengthened to effectively continue their activities before, during, and after disasters.

## Figures and Tables

**Table 1:** Demographic information of the participants.

Variable	Groups	Frequency	Percent
Gender	Woman	184	58.4
	Male	131	41.6
Marital status	Married	219	69.5
	Single	96	30.5
Status of having a child	Yes	226	71.7
	No	89	28.3
Educational Status	Primary school graduate	4	1.3
	Secondary school graduate	16	5.1
	High school graduate	92	29.2
	Associate degree graduate	47	14.9
	Bachelor’s degree graduate	126	40.0
	Degree graduate	19	6.0
	PhD graduate	11	3.5
Profession Classification	Healthcare class employees	161	51.1
	Administrative and support service class employees	154	48.9
Working time in the profession	5 years or less	66	21.0
	6–10 years	57	18.1
	11–15 years	56	17.8
	16–20 years	43	13.7
	21 years or more	93	29.5
Chronic disease status	Yes	94	29.8
	No	221	70.2
Presence of individuals with disabilities or over 80 years of age in the household for whom there are caring responsibilities	Yes	48	15.2
	No	267	84.8
Home loan payment status	Yes	72	22.9
	No	243	77.1
Place of work	Adana	15	4.8
	Adıyaman	30	9.5
	Diyarbakır	10	3.2
	Elazığ	7	2.2
	Gaziantep	34	10.8
	Hatay	37	11.7
	Kahramanmaraş	36	11.4
	Kilis	8	2.5
	Malatya	118	37.5
	Osmaniye	9	2.9
	Şanlıurfa	11	3.5
Total		315	100.0
**Variable**		**Mean ± sd**	**Min–Max**
Age		39.03 ± 9.19	22–56

sd: standard deviation.

**Table 2:** Distribution of earthquake-related variables.

Variable	Groups	Frequency	Percent
Status in terms of receiving disaster management training	Yes	117	37.1
	No	198	62.9
Loss of first- or second-degree relatives in the earthquake	Yes	49	15.6
	No	266	84.4
Status of residence	Homeowner	205	65.1
	Tenant	110	34.9
Damage to one’s house in the earthquake	Inhabitable (undamaged/slightly damaged)	207	65.7
	Moderate, heavily damaged, or destroyed	108	34.3
Current place of stay	In my current home	130	41.3
	In a car	2	0.6
	In the institution I work	24	7.6
	In a tent	30	9.5
	In a container	10	3.2
	In dormitories or other public guesthouses	10	3.2
	Outside the disaster area	25	7.9
	Next to friends or relatives	68	21.6
	Other	16	5.1
Sending family members out of the city	Yes	114	36.2
	No	190	60.3
	I live alone	11	3.5
Requesting assignment outside the disaster area	Yes	94	29.8
	No	221	70.2
Working status in the workplace after the disaster	Like before the earthquake	106	33.7
	1 day per week	106	33.7
	2 days in a week	103	32.7
Access to basic necessities after the disaster	Yes	126	40.0
	No	104	33.0
	Partially	85	27.0
Existence of compulsory earthquake insurance in the house	Yes	133	42.2
	No	182	57.8
Availability of home insurance	Yes	74	23.5
	No	241	76.5
Items retrieved from the damaged home	Yes	44	14.0
	No	50	15.9
	I was only able to retrieve some of these	44	14.0
Total		315	100.0

**Table 3:** Descriptive statistics of scale scores.

Scale	Mean ± sd	Min–Max	Cronbach’s α
Psychological hardiness	71.74 ± 11.1	25–100	0.837
Coping with stress	87.17 ± 12.66	54–120	0.903
Traumatic stress	44.04 ± 11.9	14–50	0.952
Disability	3.75 ± 2.2	0–8	0.746

sd: standard deviation.

**Table 4:** Comparison of scale scores according to demographic variables.

Variable	Groups	PHS		CRI		TSSS		DS		PETSSS	
		Mean ± sd	M (Min–Max)	Mean ± sd	M (Min–Max)	Mean ± sd	M (Min–Max)	Mean ± sd	M (Min–Max)	Mean ± sd	M (Min–Max)
Gender	Female	71.25 ± 11.06	72.5 (38–100)	85.79 ± 12.51	85 (54–120)	47.52 ± 11.4	46 (25–68)	4.26 ± 2.05	4 (0–8)	51.78 ± 12.89	50 (25–75)
	Male	72.42 ± 11.17	73 (25–96)	89.09 ± 12.66	92 (60–113)	39.16 ± 10.85	37 (19–68)	3.04 ± 2.21	2 (0–7)	42.2 ± 12.66	40 (19–75)
Mann–Whitney U		11.076		9.818		7.164		8.254		7.109	
Sig. (p)		0.220		0.005 *		0.001 *		0.001 *		0.001 *	
Profession classification	Healthcare	69.99 ± 12.36	73 (25–100)	86.49 ± 12.97	86 (54–120)	44.73 ± 10.96	43 (25–68)	3.83 ± 1.68	4 (0–7)	48.56 ± 12.06	47 (25–74)
	Administrative and support services	72.95 ± 9.99	72 (41–96)	87.63 ± 12.45	87 (58–111)	43.57 ± 12.52	43 (19–68)	3.7 ± 2.5	3 (0–8)	47.27 ± 14.62	47 (19–75)
Mann–Whitney U		10.617		11.230		11.372		11.501		11.299	
Sig. (p)		0.082		0.334		0.431		0.529		0.380	
Working time in the profession	5 years or less	71.91 ± 11.13	74.5 (38–91)	81 ± 11.63	81 (54–108)	46.18 ± 12.97	45.5 (23–68)	3.95 ± 2.18	4 (0–8)	50.14 ± 14.71	48.5 (23–75)
	6–10 years	76.74 ± 10.64	74 (62–100)	91.16 ± 13.83	89 (66–120)	46.86 ± 12.2	45 (19–68)	4.44 ± 2.24	5 (0–8)	51.3 ± 13.91	50 (19–75)
	11–15 years	71.73 ± 11.24	72 (41–91)	90.57 ± 12.13	90 (63–109)	39.93 ± 11.51	36.5 (19–68)	3.16 ± 2.16	3 (0–7)	43.09 ± 13.36	40 (19–75)
	16–20 years	72.65 ± 10.4	74 (47–88)	86.56 ± 12.64	86 (58–111)	41.21 ± 10.13	42 (26–66)	3 ± 2.3	3 (0–7)	44.21 ± 11.84	44 (26–73)
	21 years or more	68.13 ± 10.49	71 (25–83)	87.32 ± 11.39	88 (60–108)	44.59 ± 11.19	43 (23–68)	3.89 ± 2.01	4 (0–8)	48.48 ± 12.68	48 (25–75)
Kruskal–Wallis		15.670		24.118		15.655		14.740		17.079	
Sig. (p)		0.003 *		0.001 *		0.004 *		0.005 *		0.002 *	

sd: standard deviation; M: median; * *p* < 0.05: statistical disparity between groups; PHS: Psychological Hardiness Scale; CRI: Coping Responses Inventory; TSSS: Traumatic Stress Symptom Scale; DS: Disability Scale; PETSSS: Post-Earthquake Traumatic Stress Screening Scale.

**Table 5:** Correlation analysis of relationships between age and scales.

Variables		PHS	CRI	TSSS	DS	PETSSS
Age	r^1^	−0.134	0.118	−0.173	−0.148	−0.175
	*p*	0.097	0.067	0.072	0.059	0.086
PHS	r^2^		0.520	−0.281	−0.270	−0.202
	*p*		0.001 *	0.001 *	0.001 *	0.001 *
CRI	r^2^			−0.266	−0.241	−0.284
	*p*			0.003 *	0.001 *	0.001 *
TSSS	r^2^				0.749	0.994
	*p*				0.001 *	0.001 *
DS	r^2^					0.816
	*p*					0.001 *

PHS: Psychological Hardiness Scale; CRI: Coping Responses Inventory; TSSS: Traumatic Stress Symptom Scale; DS: Disability Scale; PETSSS: Post-Earthquake Traumatic Stress Screening Scale; r^1^: Spearman’s rank correlation coefficient; r^2^: Pearson’s correlation coefficient; * *p* < 0.05: statistical significance.

**Table 6:** Measurement model coefficients.

Dependent Variable	Independent Variable	β_1_	β_2_	*p*	R^2^
Traumatic stress symptoms	Psychological resilience	−0.26	−0.20	0.001 *	0.07

β_1_: standardized regression coefficients; β_2_: unstandardized regression coefficients; * *p* < 0.05: *t*-test result for the significance of the regression coefficients.

**Table 7:** Coefficients of the relationships between the scales as a result of the mediation analysis.

Dependent Variable	Independent Variable	β_1_	β_2_	*p*	R^2^
Coping with stress	Psychological resilience	0.52	0.59	0.001 *	0.27
Traumatic stress symptoms	Psychological resilience	−0.19	−0.14	0.008 *	0.08
	Coping with stress	−0.13	−0.09	0.048 *	

β_1_: standardized regression coefficients; β_2_: unstandardized regression coefficients, * *p* < 0.05: *t*-test results indicate the significance of the regression coefficients; R^2^: explanatory coefficients.

## Data Availability

These data are kept confidential within the scope of personal data protection law. However, upon reasonable request, the data can be accessed by contacting the corresponding author.
